# Deep Learning-Based Recognition of Periodontitis and Dental Caries in Dental X-ray Images

**DOI:** 10.3390/bioengineering10080911

**Published:** 2023-08-01

**Authors:** Ivane Delos Santos Chen, Chieh-Ming Yang, Mei-Juan Chen, Ming-Chin Chen, Ro-Min Weng, Chia-Hung Yeh

**Affiliations:** 1 Department of Electrical Engineering, National Dong Hwa University, Hualien 97401, Taiwan; 2 Department of Electrical Engineering, National Taiwan Normal University, Taipei 10610, Taiwan; 3 Department of Electrical Engineering, National Sun Yat-sen University, Kaohsiung 80424, Taiwan

**Keywords:** periodontitis, dental caries, dental X-ray, deep learning, YOLOv7, convolutional neural network

## Abstract

Dental X-ray images are important and useful for dentists to diagnose dental diseases. Utilizing deep learning in dental X-ray images can help dentists quickly and accurately identify common dental diseases such as periodontitis and dental caries. This paper applies image processing and deep learning technologies to dental X-ray images to propose a simultaneous recognition method for periodontitis and dental caries. The single-tooth X-ray image is detected by the YOLOv7 object detection technique and cropped from the periapical X-ray image. Then, it is processed through contrast-limited adaptive histogram equalization to enhance the local contrast, and bilateral filtering to eliminate noise while preserving the edge. The deep learning architecture for classification comprises a pre-trained EfficientNet-B0 and fully connected layers that output two labels by the sigmoid activation function for the classification task. The average precision of tooth detection using YOLOv7 is 97.1%. For the recognition of periodontitis, the area under the curve (AUC) of the receiver operating characteristic (ROC) curve is 98.67%, and the AUC of the precision-recall (PR) curve is 98.38%. For the recognition of dental caries, the AUC of the ROC curve is 98.31%, and the AUC of the PR curve is 97.55%. Different from the conventional deep learning-based methods for a single disease such as periodontitis or dental caries, the proposed approach can provide the recognition of both periodontitis and dental caries simultaneously. This recognition method presents good performance in the identification of periodontitis and dental caries, thus facilitating dental diagnosis.

## 1. Introduction

With the continuous improvement of artificial intelligence (AI) technology and big data availability, applications in medical imaging research are increasingly emphasized [1]. AI has shown great potential to assist in disease diagnosis and treatment planning in dentistry [2,3,4]. Deep learning models have demonstrated outstanding abilities in learning complex patterns from large image datasets, giving rise to numerous applications in the field of dentistry [2,5,6,7,8,9]. Deep learning of dental radiographs has emerged as an efficient and precise method for detecting dental diseases. By applying the convolutional neural network, an effective system can be established for the recognition of dental diseases.

Oral disease is an important problem of global public health, especially common dental diseases such as periodontitis and dental caries. Periodontitis, a chronic inflammatory disease of the teeth and gums, is characterized by the destruction of surrounding tissues including the periodontal ligament and the alveolar bone. Periodontitis is mainly caused by dental plaque, which produces a series of inflammatory reactions and destroys periodontal tissues. It is found that periodontitis may increase the risk of cardiovascular disease or be related to other major systemic diseases [10]. Dental caries is a disease that can damage tooth structure and is mainly caused by acid erosion. The acid is mostly produced by intraoral bacteria. Periodontitis and dental caries are the main oral diseases with high prevalence that influence the quality of life [11]. Periodontitis and dental caries are two of the most prevalent oral diseases globally, affecting a significant proportion of the population. These two diseases can have a profound impact on oral health and overall well-being. Therefore, the prevention of periodontitis and dental caries is an important task for dentists. Moreover, early diagnosis has always been a crucial part of the treatment of periodontitis and dental caries. The detection of periodontitis and dental caries mainly depends on clinical and radiographic examinations. Dentists typically evaluate various aspects of a patient’s oral condition to provide a comprehensive diagnosis and treatment plan. Using AI-assisted technology to concurrently recognize both periodontitis and dental caries aligns closely with how dentists diagnose patients in clinical practice. Saving time and reducing loading for dentists and minimizing patients’ discomfort associated with multiple examinations can be realized.

The applications of AI to dental X-ray images can assist dentists in quickly and accurately identifying common dental diseases. The related work on the applications of AI for dental X-rays is discussed below.

A deep learning-based convolutional neural network (CNN) algorithm was developed in [12] for the predictions of periodontally compromised teeth (PCT) for premolars and molars individually. The study used 16 convolutional layers and 3 fully connected dense layers in the deep CNN model to classify the teeth into healthy teeth, moderate PCT, and severe PCT. In [13], a vector of the severity of alveolar bone loss from the teeth was used as the input feature of XGBoost to classify the four-class severity degree of periodontitis from a panoramic radiograph. In [14], periapical radiographs were used to calculate the radiographic bone loss (RBL) values and classify the severity of RBL into mild or severe, as well as classify the defect morphology. These two tasks were performed by a multi-task classification approach using the InceptionV3 model.

In [15], the modified linearly adaptive particle swarm optimization was combined with a backpropagation neural network to distinguish between normal and caries affected teeth. In [16], the prediction of dental caries of premolars and molars was based on a pre-trained GoogLeNet InceptionV3 CNN network for preprocessing and transfer learning. In [17], a system for predicting dental caries was developed using Laplacian filtering, window-based adaptive thresholding, morphology, statistical features, and a backpropagation neural network. In [18], Hu’s moment was used to train support vector machine and k-nearest neighbors for the classification of four levels of dental caries. In [19], both raw periapical images and the enhanced images were the inputs of an ensemble deep convolutional neural network model for dental caries detection. In [20], informative features were extracted from teeth on panoramic radiographs via deep learning networks, and each extracted feature set was used to train the classification model. The caries screening was determined by a majority voting method.

In [21], deep convolutional neural networks with region proposal techniques were used to detect decay, periapical periodontitis, and periodontitis on periapical radiographs. The three diseases were individually classified into mild, moderate, and severe levels. In [22], the periapical radiograph subregion was cropped to obtain a single-tooth image. Then, the crown region and the root region were cropped according to the identified cervical line. The detection of caries from the crown region and periapical periodontitis from the root region was based on a deep learning model constructed of two cascaded ResNet-18 backbones and two individual convolutional layers.

Most of the studies on AI-assisted technology for dental diseases are for the prediction of a single disease such as periodontitis or dental caries. This paper proposes a deep learning-based method to detect periodontitis and dental caries simultaneously. The image processing technologies are also incorporated to improve performance.

## 2. Materials and Methods

**Figure 1:**
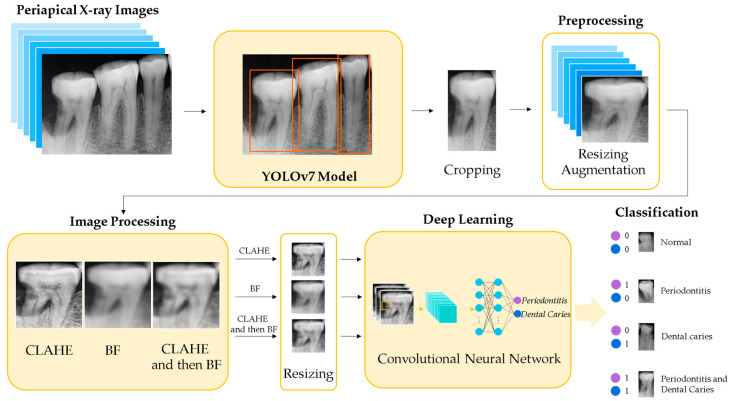
Flowchart of the training process for the proposed method.

Figure 1 shows the flowchart of the training process for the proposed method to detect periodontitis and dental caries simultaneously. The single-tooth X-ray images are detected by the YOLOv7 [23] algorithm and cropped from periapical X-ray images. After performing resizing and augmentation, the single-tooth X-ray images are enhanced by contrast-limited adaptive histogram equalization (CLAHE) and bilateral filtering (BF). The enhanced images are further resized as the inputs for the deep-learning CNN, which is trained using transfer learning to determine whether the single-tooth X-ray image belongs to normal tooth, periodontitis, dental caries, or both diseases of periodontitis and dental caries.

### 2.1. Dataset

A total of 1525 periapical X-ray images were obtained from a dental clinic in Hualien, Taiwan. In periapical X-ray images, periodontitis can be detected by the presence of alveolar bone loss around the tooth. Dental caries can be recognized by the radiolucency of enamel and dentin in the tooth structure. In this study, a normal tooth was defined as when the characteristics of periodontitis and dental caries are not detected in the X-ray image. Both anterior and posterior teeth are included in our dataset. The teeth with root canal therapy and dental restoration are included. The teeth on periapical X-ray images were annotated by a senior dentist with over 28 years of expertise. This study used two labels of periodontitis and dental caries to classify a single-tooth X-ray image belonging to normal tooth, periodontitis, dental caries, or both diseases. Each label is 0 or 1, and thus the corresponding classification of the single-tooth X-ray image is normal (0,0), periodontitis-only (1,0), dental caries-only (0,1), or both diseases of periodontitis and dental caries (1,1).

The single-tooth X-ray images were cropped from periapical X-ray images by the YOLOv7 object detection model. A total of 2850 single-tooth X-ray images were selected in the experiments and resized to 200 × 200 pixels. Data augmentation was used to increase the number of images. The augmentation for single-tooth images included horizontal flip, vertical flip, and the rotations of 90°, 180°, and 270°. The total dataset was divided into the training dataset (n = 8000), the validation dataset (n = 2000), and the testing dataset (n = 1000), as listed in Table 1. This study used 10-fold cross-validation by randomly dividing the dataset into ten subsets to evaluate the performance of the deep learning models.

### 2.2. Proposed Method

Table 2 presents a comprehensive overview of the hardware and software platforms employed in the experimental setup. The hardware platform consists of a 12th Gen Intel Core i5-12400 CPU, an NVIDIA GeForce RTX 3070 GPU, and 32 GB DDR4 DRAM with 3200 MHz. On the software side, the platform includes Python version 3.7.16, Tensorflow version 2.9.1, and PyTorch version 1.7.1. These specifications are employed to facilitate the implementation and evaluation of the proposed methods in our study.

This study used YOLOv7 to obtain single-tooth images from periapical X-ray images to avoid the time-consuming task of manual cropping. YOLO (you only look once) is a classic one-stage, real-time object detection system known for its lightweight, low dependency, and highly efficient algorithm. Compared to two-stage object detection, the one-stage approach eliminates the need for candidate pre-screening, enabling end-to-end object detection and obtaining the final classification in a single pass. YOLO has demonstrated superior performance in object detection compared to other algorithms [24]. YOLOv7 has achieved remarkable advancements in both accuracy and processing speed [23]. The remarkable advancements make it well-suited for precise and fast detection to obtain single-tooth images from periapical X-ray images. To train the YOLOv7 model, the batch size of 8 and the epoch of 50 were selected to make a trade-off between computational resources and training accuracy.

**Figure 2:**
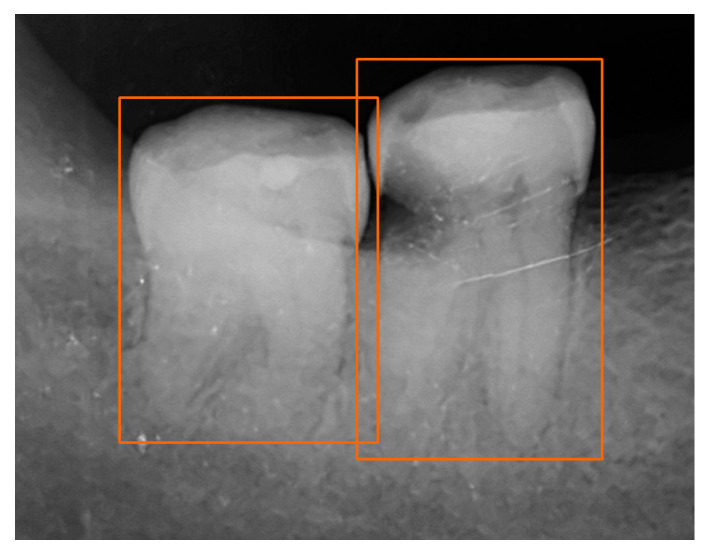
Detection results by YOLOv7.

**Figure 3:**
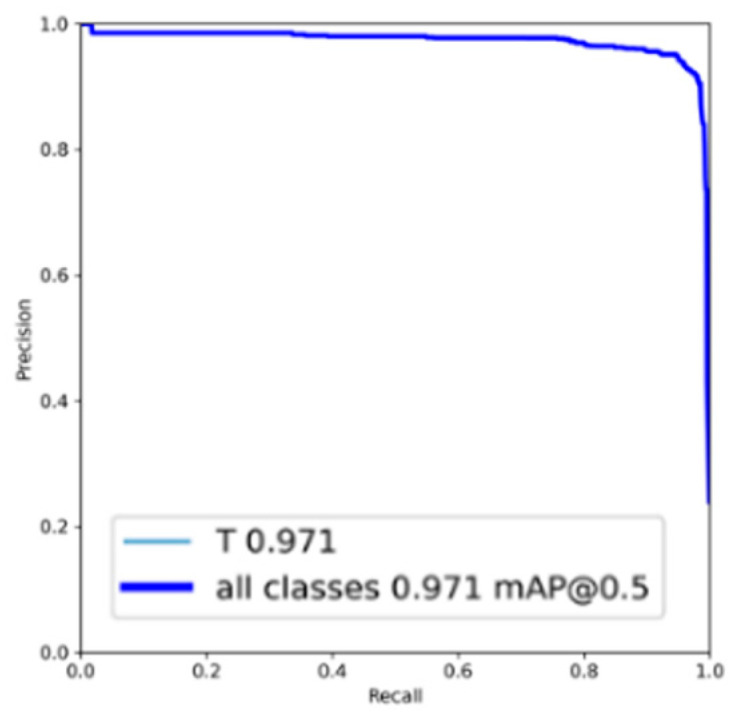
PR curve of YOLOv7 detection.

In Figure 2, an illustrative example of single-tooth image detection from a periapical X-ray image is depicted. It can be observed that YOLOv7 demonstrates precise localization by accurately bounding the position of each tooth. Figure 3 shows the PR curve derived from the predictions made by YOLOv7 on the testing dataset. Notably, the average precision (AP) achieved an outstanding performance of 97.1% for tooth detection, manifesting its excellent effectiveness.

In addition to utilizing YOLOv7 for single-tooth image detection in periapical X-ray images, image enhancement techniques have been incorporated to enhance the performance of deep learning algorithms. Contrast-limited adaptive histogram equalization (CLAHE) can enhance the local details of images [25,26]. The histogram of the local area is calculated to redistribute the image brightness. The bilateral filter (BF) [27] is a non-linear filter for smoothing images while preserving edge information because not only the geometric distance between pixels but also the difference of gray-level values between pixels is considered. CLAHE and BF have been used for the preprocessing of the segmentation of teeth in dental radiographs [28].

**Figure 4:**
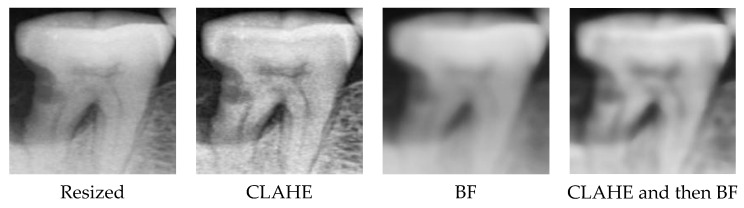
Illustration of the image processing techniques.

After performing resizing and augmentation of the cropped single-tooth X-ray images, the images are processed using CLAHE, BF, and the combination of CLAHE first and then BF, as illustrated in Figure 4. CLAHE is mainly used to enhance the local contrast of the X-ray image. After increasing the contrast, there may be some detailed noises. Then, the BF blurs the less relevant areas and reduces the noise but preserves the edge of the image. CLAHE can make the tooth outline sharper and help to reveal subtle details hidden due to low contrast. The BF further reduces noise in the image, yielding a smoother appearance while preserving the overall structure and contour of the tooth. The two image processing techniques can facilitate the feature extraction of CNN and improve the prediction performance of deep learning. The processed images are further resized to 100 × 100 as the inputs of the CNN model.

CNN model can be utilized to build a multi-label classifier for the prediction of periodontitis and dental caries. In this study, we used Xception [29], MobileNetV2 [30], and EfficientNet-B0 [31] to compare their performances and select the best one. The deep learning architecture for classification consists of the pre-trained CNN model and fully connected layers that output two labels by the sigmoid activation function for the multi-label classification task. Transfer learning is applied by initializing the models with pre-trained weights provided by Keras during the training process. Furthermore, Table 3 presents an overview of the hyperparameters employed specifically in the CNN models. By adjusting these hyperparameters, better performance can be achieved.

### 2.3. Performance Metrics

To evaluate the performance of the proposed method, various metrics are used, including accuracy, sensitivity, specificity, positive predictive value (*PPV*, precision), negative predictive value (*NPV*), the area under the curve (AUC) of the receiver operating characteristic (ROC) curve, and the AUC of precision–recall (PR) curve. Accuracy, sensitivity, specificity, *PPV*, and *NPV* are defined by true positive (*TP*), false negative (*FN*), true negative (*TN*), and false positive (*FP*), as represented by Equations (1)–(5).

(1) $$Accuracy=\frac{TP+TN}{TP+TN+FP+FN},$$

(2) $$Sensitivity\;\left(Recall\right)=\frac{TP}{TP+FN},$$

(3) $$Specificity=\frac{TN}{TN+FP},$$

(4) $$PPV\;\left(Precision\right)=\frac{TP}{TP+FP},$$

(5) $$NPV=\frac{TN}{TN+FN}.$$

## 3. Results

We compared the performances of three CNN models through a 10-fold cross-validation analysis. The investigated models included Xception, MobileNetV2, and EfficientNet-B0. Each model was trained using pre-trained weights provided by Keras for transfer learning. Table 4 tabulates the results of performance metrics averaged from 10-fold cross-validation for the various models. Notably, EfficientNet-B0 achieved the best result with the accuracy of 95.44%, the sensitivity of 93.28%, the specificity of 96.88%, the PPV of 95.24%, and the NPV of 95.59% for periodontitis, and the accuracy of 94.94%, the sensitivity of 94.15%, the specificity of 95.47%, the PPV of 93.30%, and the NPV of 96.08% for dental caries.

Table 5 lists the minimum, maximum, and mean accuracy rates of each model across 10-fold cross-validation. Notably, EfficientNet-B0 demonstrated the highest mean accuracy, achieving 95.44% for periodontitis and 94.94% for dental caries. These metrics indicate that EfficientNet-B0 outperforms the other models evaluated in this study. Thus, EfficientNet-B0 was chosen as the CNN model in our method.

Table 6 provides the average evaluation metrics for each fold using EfficientNet-B0, demonstrating the performance of the model across different subsets of the dataset. It can be observed that the predictive accuracy rates for periodontitis and dental caries are consistently above 92% for all the subsets in the 10-fold cross-validation. These findings indicate the robust performance and strong predictive capabilities of the EfficientNet-B0 model in accurately classifying periodontitis and dental caries.

**Figure 5:**
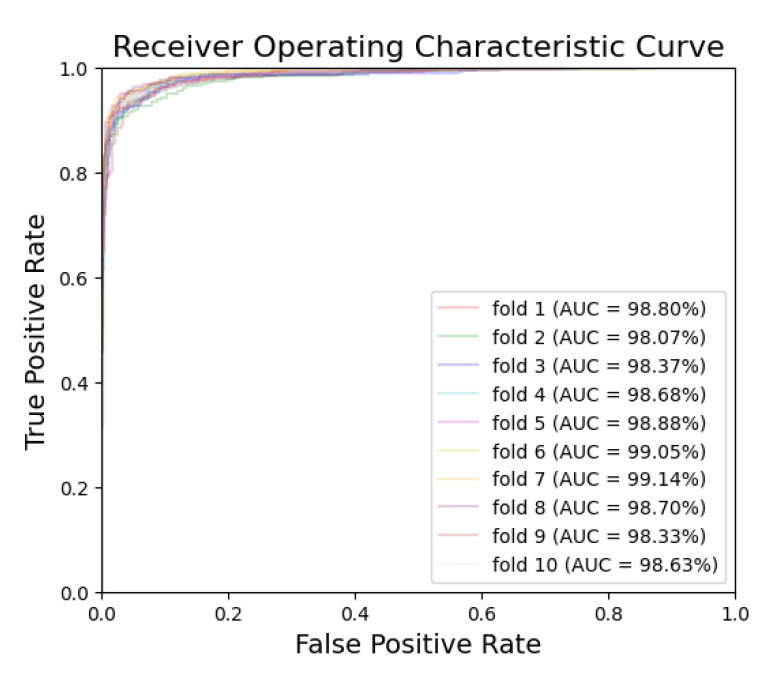
ROC curve for periodontitis.

**Figure 6:**
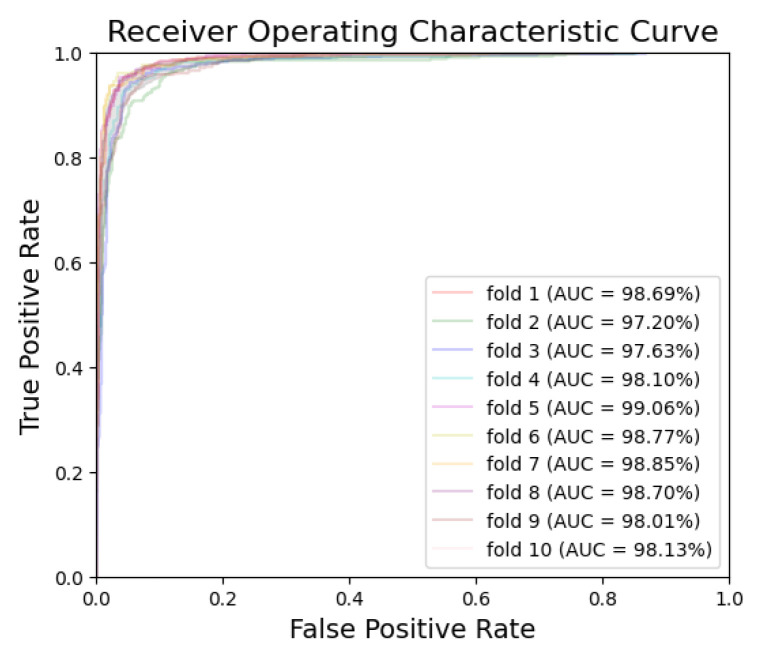
ROC curve for dental caries.

**Figure 7:**
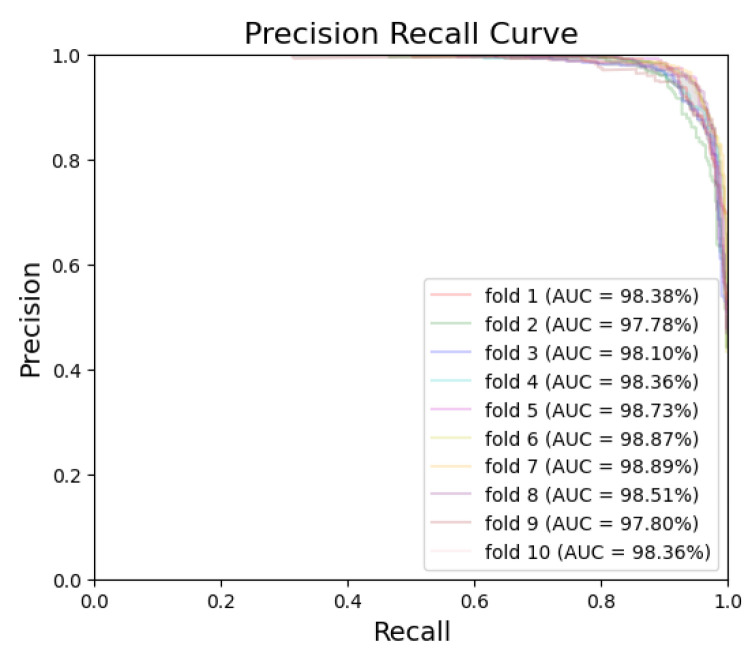
PR curve for periodontitis.

**Figure 8:**
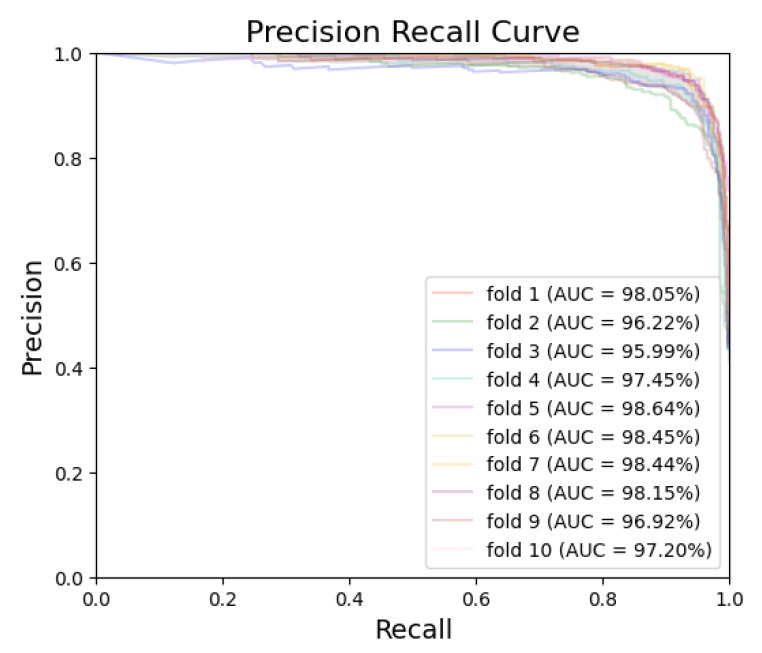
PR curve for dental caries.

Figure 5 and Figure 6 exhibit the ROC curves for periodontitis and dental caries, respectively, in each fold. The utilization of the EfficientNet-B0 model shows remarkable performance for periodontitis and dental caries, with the AUC values reaching 98.67% and 98.31%, respectively. Furthermore, Figure 7 and Figure 8 illustrate the PR curves for periodontitis and dental caries, respectively, in each fold. These curves further emphasize the excellent performance of the EfficientNet-B0 model, with the AUC value of 98.38% for periodontitis and 97.55% for dental caries.

We conducted an ablation study to examine the impact of image processing techniques on the performance of the EfficientNet-B0 model. The results, shown in Table 7, demonstrate that incorporating image processing techniques improves the performance in recognizing periodontitis and dental caries. With image processing, the model achieved higher accuracy. Additionally, there were improvements in sensitivity, specificity, PPV, and NPV.

Moreover, the implementation of image processing techniques resulted in enhancements in both the ROC AUC and the PR AUC. For the prediction of periodontitis, the ROC AUC was improved from 96.72% to 98.67%, and the PR AUC increased from 96.22% to 98.38%. Similarly, in the case of dental caries prediction, the ROC AUC was enhanced from 96.49% to 98.31%, and the PR AUC increased from 95.83% to 97.55%. Overall, image processing is efficient to improve the prediction performance of two labels from all metrics.

**Figure 9:**
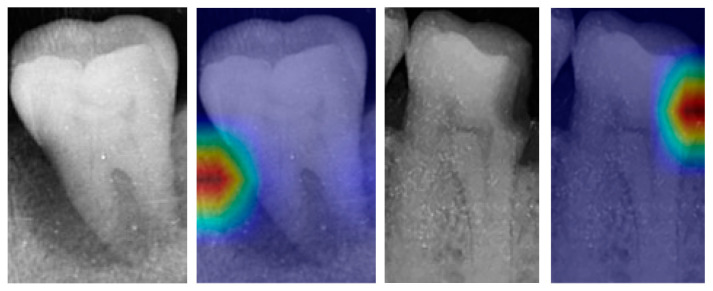
Illustration based on Grad-CAM.

To improve the interpretability of the EfficientNetB0 model, the illustration utilizing gradient-weighted class activation mapping (Grad-CAM) [32] is shown in Figure 9. The examples, by superimposing the up-sampled heat maps over the single-tooth X-ray images, effectively highlight the active regions within the images. These regions exert great influence on the classification results of the EfficientNet-B0 model. Figure 9 provides valuable insights into the decision-making mechanism.

## 4. Discussion

The study in [16] focused on detecting dental caries on one tooth per cropped image based on deep learning for premolars and molars in periapical radiographs. The accuracy, sensitivity, specificity, PPV, NPV, and ROC AUC for the model of predicting dental caries for both premolars and molars in [16] were 82.0%, 81.0%, 83.0%, 82.7%, 81.4%, and 84.5%, respectively. Our method for predicting dental caries provided the accuracy of 94.94%, the sensitivity of 94.15%, the specificity of 95.47%, the PPV of 93.30%, the NPV of 96.08%, and the ROC AUC of 98.31%, which outperforms [16], as shown in Table 8. In particular, our method can recognize both periodontitis and dental caries simultaneously. In addition, both anterior and posterior teeth are included in our dataset.

For a single tooth, the deep learning model proposed in [22] needs to be executed twice to obtain the detection of periapical periodontitis and caries. The first time is to obtain the dental root result for the detection of periapical periodontitis and the second time is to acquire the dental crown results for the detection of caries. However, the multi-label deep learning architecture of our method only needs to be executed once to obtain the recognition results among the four kinds of teeth. In [22], the sensitivity, specificity, PPV, NPV, and ROC AUC for the performance of periapical periodontitis were 82.00%, 84.00%, 83.67%, 82.35%, and 87.90%, respectively. Our model performed with the sensitivity of 93.28%, the specificity of 96.88%, the PPV of 95.24%, the NPV of 95.59%, and the ROC AUC of 98.67% for periodontitis. For the prediction of dental caries, the sensitivity, specificity, PPV, NPV, and ROC AUC were 83.50%, 82.00%, 82.27%, 83.25%, and 87.50%, respectively, in [22]; however, they were 94.15%, 95.47%, 93.30%, 96.08%, and 98.31%, respectively, in our method, as shown in Table 9. In addition, cropping a single tooth is not a fully automated method in [22]. Our method provides an automated tooth detection process using YOLOv7.

The automatic evaluation of periodontitis and dental caries can facilitate the initial screening of dental conditions, particularly benefiting people in underserved areas with limited access to healthcare resources. It can promote regular dental check-ups, thereby contributing to the overall maintenance of oral health. Additionally, the screening results obtained through this method can assist healthcare decision-makers in investigating healthcare demands, optimizing the allocation of dental workforces, and effectively reducing the urban–rural disparity in dental care accessibility. The simultaneous recognition method has the potential to positively impact public health strategies, healthcare planning, and the overall accessibility of dental care, especially for medically underserved populations.

This study focused solely on dental X-ray images, which may exclude important clinical factors, such as the patient’s medical and dental history, symptoms, and other clinical examination results. These clinical factors provide valuable information that can contribute to the overall assessment of the patient’s condition. The exclusion of these clinical factors could limit the performance of the proposed method. Therefore, future research is suggested to evaluate both dental X-ray images and clinical factors before annotation and training AI models. This integration can potentially enhance the accuracy and effectiveness of AI-assisted technology.

## 5. Conclusions

Artificial intelligence technologies have made significant progress recently in the applications of dentistry. This paper presents an effective method for the simultaneous recognition of periodontitis and dental caries in dental X-ray images. The methodology applies YOLOv7 for tooth detection, image processing technologies (contrast-limited adaptive histogram equalization and bilateral filtering), and the EfficientNet-B0 model. The proposed method achieved good performance in terms of various performance metrics. This deep learning-based method, which demonstrated promising capabilities, can be beneficial to dentists for the identification of periodontitis and dental caries.

## Figures and Tables

**Table 1:** Numbers of single-tooth X-ray images in the experiments.

	Dataset	Normal	Periodontitis	Dental Caries	Both Diseases	Total
Original	Training and Validation	700	1000	400	500	2600
	Testing	100	50	50	50	250
Augmentation	Training and Validation	3300	1000	1600	1500	7400
	Testing	300	150	150	150	750
Total	Training	3200	1600	1600	1600	8000
	Validation	800	400	400	400	2000
	Testing	400	200	200	200	1000

**Table 2:** Hardware and software platforms.

**Hardware Platform**	**Version**
CPU	12th Gen Intel Core i5-12400
GPU	NVIDIA GeForce RTX 3070
DRAM	32 GB DDR4 3200 MHz
**Software Platform**	**Version**
Python	3.7.16
Tensorflow	2.9.1
PyTorch	1.7.1

**Table 3:** Hyperparameters in the CNN models.

Hyperparameter	Value
Initial learning rate	0.001
Max epoch	50
Batch size	50
Learning drop period	4
Learning rate drop factor	0.316

**Table 4:** Performance comparison of the various CNN models.

Model	Disease	Accuracy	Sensitivity	Specificity	PPV	NPV	ROC AUC	PR AUC
		(%)	(%)	(%)	(%)	(%)	(%)	(%)
Xception	Periodontitis	89.76	89.26	90.59	86.40	92.54	94.58	93.34
	Dental caries	88.13	86.98	88.41	83.50	91.21	93.49	90.44
MobileNetV2	Periodontitis	91.42	91.21	91.60	87.73	94.01	96.86	95.89
	Dental caries	89.03	88.25	89.51	85.32	91.87	96.31	94.76
EfficientNet-B0	Periodontitis	95.44	93.28	96.88	95.24	95.59	98.67	98.38
	Dental caries	94.94	94.15	95.47	93.30	96.08	98.31	97.55

**Table 5:** Accuracy comparison of the various CNN models.

Model	Periodontitis			Dental Caries		
	Minimum (%)	Maximum (%)	Mean (%)	Minimum (%)	Maximum (%)	Mean (%)
Xception	88.98	91.66	89.76	86.89	89.11	88.13
MobileNetV2	89.98	92.51	91.42	87.36	90.42	89.03
EfficientNet-B0	94.60	96.30	95.44	92.80	96.40	94.94

**Table 6:** Performance of EfficientNet-B0 on 10-fold cross-validation.

	Disease	TP	FN	TN	FP	Accuracy	Sensitivity	Specificity	PPV	NPV	ROC AUC	PR AUC
						(%)	(%)	(%)	(%)	(%)	(%)	(%)
Fold-1	Periodontitis	369	31	586	14	95.50	92.25	97.67	96.34	94.98	98.80	98.38
	Dental caries	381	19	574	26	95.50	95.25	95.67	93.61	96.80	98.69	98.05
Fold-2	Periodontitis	361	39	585	15	94.60	90.25	97.50	96.01	93.75	98.07	97.78
	Dental caries	363	37	565	35	92.80	90.75	94.17	91.21	93.85	97.20	96.22
Fold-3	Periodontitis	369	31	580	20	94.90	92.25	96.67	94.86	94.93	98.37	98.10
	Dental caries	377	23	568	32	94.50	94.25	94.67	92.18	96.11	97.63	95.99
Fold-4	Periodontitis	371	29	576	24	94.70	92.75	96.00	93.92	95.21	98.68	98.36
	Dental caries	372	28	572	28	94.40	93.00	95.33	93.00	95.33	98.10	97.45
Fold-5	Periodontitis	380	20	582	18	96.20	95.00	97.00	95.48	96.68	98.88	98.73
	Dental caries	381	19	579	21	96.00	95.25	96.50	94.78	96.82	99.06	98.64
Fold-6	Periodontitis	376	24	583	17	95.90	94.00	97.17	95.67	96.05	99.05	98.87
	Dental caries	384	16	580	20	96.40	96.00	96.67	95.05	97.32	98.77	98.45
Fold-7	Periodontitis	377	23	586	14	96.30	94.25	97.67	96.42	96.22	99.14	98.89
	Dental caries	375	25	588	12	96.30	93.75	98.00	96.90	95.92	98.85	98.44
Fold-8	Periodontitis	382	18	575	25	95.70	95.50	95.83	93.86	96.96	98.70	98.51
	Dental caries	378	22	578	22	95.60	94.50	96.33	94.50	96.33	98.70	98.15
Fold-9	Periodontitis	371	29	575	25	94.60	92.75	95.83	93.69	95.20	98.33	97.80
	Dental caries	379	21	557	43	93.60	94.75	92.83	89.81	96.37	98.01	96.92
Fold-10	Periodontitis	375	25	585	15	96.00	93.75	97.50	96.15	95.90	98.63	98.36
	Dental caries	376	24	567	33	94.30	94.00	94.50	91.93	95.94	98.13	97.20
Mean	Periodontitis	--	--	--	--	95.44	93.28	96.88	95.24	95.59	98.67	98.38
	Dental caries	--	--	--	--	94.94	94.15	95.47	93.30	96.08	98.31	97.55

**Table 7:** Ablation study.

Method	Disease	Accuracy (%)	Sensitivity (%)	Specificity (%)	PPV (%)	NPV (%)	ROC AUC (%)	PR AUC (%)
With image processing	Periodontitis	95.44	93.28	96.88	95.24	95.59	98.67	98.38
	Dental caries	94.94	94.15	95.47	93.30	96.08	98.31	97.55
Without image processing	Periodontitis	93.05	92.10	93.68	90.77	94.72	96.72	96.22
	Dental caries	92.91	92.33	93.30	90.23	94.80	96.49	95.83

**Table 8:** Comparison of the proposed method with [16].

Method	CNN Network	Accuracy (%)	Sensitivity (%)	Specificity (%)	PPV (%)	NPV (%)	ROC AUC (%)
[16]	GoogLeNet InceptionV3	82.0	81.0	83.0	82.7	81.4	84.5
Proposed method	EfficientNet-B0	94.94	94.15	95.47	93.30	96.08	98.31

**Table 9:** Comparison of the proposed method with [22].

Method	CNN Network	Disease	Sensitivity (%)	Specificity (%)	PPV (%)	NPV (%)	ROC AUC (%)
[22]	Modified ResNet-18 Backbone	Periapical periodontitis	82.00	84.00	83.67	82.35	87.90
		Dental caries	83.50	82.00	82.27	83.25	87.50
Proposed method	EfficientNet-B0	Periodontitis	93.28	96.88	95.24	95.59	98.67
		Dental caries	94.15	95.47	93.30	96.08	98.31

## Data Availability

Data is unavailable due to ethical restrictions.

## References

[1] Panayides A.S., Amini A., Filipovic N.D., Sharma A., Tsaftaris S.A., Young A., Foran D., Do N., Golemati S., Kurc T. (2020). AI in medical imaging informatics: Current challenges and future directions. IEEE J. Biomed. Health Inform..

[2] Kishimoto T., Goto T., Matsuda T., Iwawaki Y., Ichikawa T. (2022). Application of artificial intelligence in the dental field: A literature review. J. Prosthodont. Res..

[3] Suhail Y., Upadhyay M., Chhibber A., Kshitiz (2020). Machine learning for the diagnosis of orthodontic extractions: A computational analysis using ensemble learning. Bioengineering.

[4] Schwendicke F., Golla T., Dreher M., Krois J. (2019). Convolutional neural networks for dental image diagnostics: A scoping review. J. Dent..

[5] Rao R.S., Shivanna D.B., Lakshminarayana S., Mahadevpur K.S., Alhazmi Y.A., Bakri M.M.H., Alharbi H.S., Alzahrani K.J., Alsharif K.F., Banjer H.J. (2022). Ensemble deep-learning-based prognostic and prediction for recurrence of sporadic odontogenic keratocysts on hematoxylin and eosin stained pathological images of incisional biopsies. J. Pers. Med..

[6] Murata M., Ariji Y., Ohashi Y., Kawai T., Fukuda M., Funakoshi T., Kise Y., Nozawa M., Katsumata A., Fujita H. (2019). Deep-learning classification using convolutional neural network for evaluation of maxillary sinusitis on panoramic radiography. Oral Radiol..

[7] Celik M.E. (2022). Deep learning based detection tool for impacted mandibular third molar teeth. Diagnostics.

[8] Chuo Y., Lin W.M., Chen T.Y., Chan M.L., Chang Y.S., Lin Y.R., Lin Y.J., Shao Y.H., Chen C.A., Chen S.L. (2022). A high-accuracy detection system: Based on transfer learning for apical lesions on periapical radiograph. Bioengineering.

[9] Chen Y.C., Chen M.Y., Chen T.Y., Chan M.L., Huang Y.Y., Liu Y.L., Lee P.T., Lin G.J., Li T.F., Chen C.A. (2023). Improving dental implant outcomes: CNN-based system accurately measures degree of peri-implantitis damage on periapical film. Bioengineering.

[10] Falcao A., Bullón P. (2019). A review of the influence of periodontal treatment in systemic diseases. Periodontol. 2000.

[11] Watt R.G., Heilmann A., Listl S., Peres M.A. (2016). London charter on oral health inequalities. J. Dent. Res..

[12] Lee J.H., Kim D.H., Jeong S.N., Choi S.H. (2018). Diagnosis and prediction of periodontally compromised teeth using a deep learning-based convolutional neural network algorithm. J. Periodontal Implant Sci..

[13] Li H., Zhou J., Zhou Y., Chen Q., She Y., Gao F., Xu Y., Chen J., Gao X. (2021). An interpretable computer-aided diagnosis method for periodontitis from panoramic radiographs. Front. Physiol..

[14] Chang J., Chang M.F., Angelov N., Hsu C.Y., Meng H.W., Sheng S., Glick A., Chang K., He Y.R., Lin Y.B. (2022). Application of deep machine learning for the radiographic diagnosis of periodontitis. Clin. Oral Investig..

[15] Sornam M., Prabhakaran M. A new linear adaptive swarm intelligence approach using back propagation neural network for dental caries classification. Proceedings of the 2017 IEEE International Conference on Power, Control, Signals and Instrumentation Engineering.

[16] Lee J.H., Kim D.H., Jeong S.N., Choi S.H. (2018). Detection and diagnosis of dental caries using a deep learning-based convolutional neural network algorithm. J. Dent..

[17] Geetha V., Aprameya K.S., Hinduja D.M. (2020). Dental caries diagnosis in digital radiographs using back-propagation neural network. Health Inf. Sci. Syst..

[18] Jusman Y., Anam M.K., Puspita S., Saleh E. Machine learnings of dental caries images based on Hu moment invariants features. Proceedings of the 2021 International Seminar on Application for Technology of Information and Communication.

[19] Imak A., Celebi A., Siddique K., Turkoglu M., Sengur A., Salam I. (2022). Dental caries detection using score-based multi-input deep convolutional neural network. IEEE Access.

[20] Bui T.H., Hamamoto K., Paing M.P. (2022). Automated caries screening using ensemble deep learning on panoramic radiographs. Entropy.

[21] Chen H., Li H., Zhao Y., Zhao J., Wang J. (2021). Dental disease detection on periapical radiographs based on deep convolutional neural networks. Int. J. Comput. Assist. Radiol. Surg..

[22] Li S., Liu J., Zhou Z., Zhou Z., Wu X., Li Y., Wang S., Liao W., Ying S., Zhao Z. (2022). Artificial intelligence or caries and periapical periodontitis detection. J. Dent..

[23] Wang C.Y., Bochkovskiy A., Liao H.Y.M. YOLOv7: Trainable bag-of-freebies sets new state-of-the-art for real-time object detectors. Proceedings of the 2023 IEEE/CVF Conference on Computer Vision and Pattern Recognition.

[24] Srivastava S., Divekar A.V., Anilkumar C., Naik I., Kulkarni V., Pattabiraman V. (2021). Comparative analysis of deep learning image detection algorithms. J. Big Data.

[25] Pizer S.M., Amburn E.P., Austin J.D., Cromartie R., Geselowitz A., Greer T., Romeny B.H., Zimmerman J.B., Zuiderveld K. (1987). Adaptive histogram equalization and its variations. Comput. Vis. Graph. Image Process..

[26] Pizer S.M., Johnston R.E., Ericksen J.P., Yankaskas B.C., Muller K.E. Contrast-limited adaptive histogram equalization: Speed and effectiveness. Proceedings of the First Conference on Visualization in Biomedical Computing.

[27] Tomasi C., Manduchi R. Bilateral filtering for gray and color images. Proceedings of the Sixth International Conference on Computer Vision.

[28] Pandey P., Bhan A., Dutta M.K., Travieso C.M. Automatic image processing based dental image analysis using automatic gaussian fitting energy and level sets. Proceedings of the 2017 International Conference and Workshop on Bioinspired Intelligence.

[29] Chollet F. Xception: Deep learning with depthwise separable convolutions. Proceedings of the 2017 IEEE Conference on Computer Vision and Pattern Recognition.

[30] Sandler M., Howard A., Zhu M., Zhmoginov A., Chen L.C. Mobilenetv2: Inverted residuals and linear bottlenecks. Proceedings of the 2018 IEEE/CVF Conference on Computer Vision and Pattern Recognition.

[31] Tan M., Le Q.V. Efficientnet: Rethinking model scaling for convolutional neural networks. Proceedings of the 36th Interna-tional Conference on Machine Learning.

[32] Selvaraju R.R., Cogswell M., Das A., Vedantam R., Parikh D., Batra D. Grad-CAM: Visual explanations from deep networks via gradient-based localization. Proceedings of the 2017 IEEE International Conference on Computer Vision.

